# Melt processing of polypropylene-grafted-maleic anhydride/Chitosan polymer blend functionalized with montmorillonite for the removal of lead ions from aqueous solutions

**DOI:** 10.1038/s41598-019-57079-2

**Published:** 2020-01-14

**Authors:** T. N. Moja, N. Bunekar, S. B. Mishra, T.-Y. Tsai, S. S. Hwang, A. K Mishra

**Affiliations:** 1 Nanotechnology and Water Sustainability Research Unit, College of Science, Engineering and Technology, University of South Africa, Florida Campus, Johannesburg, South Africa; 2 Department of Mechanical Engineering, Chien-Hsin University of Science and Technology, Chung-Li, 32097 Taiwan, ROC; 3 Department of Chemistry, Master Program in Nanotechnology & Center for Nanotechnology, Chung Yuan Christian University, Chung-Li, 32023 Taiwan, ROC

**Keywords:** Synthesis and processing, Synthesis and processing, Synthesis and processing, Synthesis and processing

## Abstract

Heavy metals such as lead ions Pb (II) are a primary concern in the aquatic environment. These is because Pb (II) is poisonous at a threshold limit above 0.01 mg/L, when consumed over a long period of time. Pb (II) poisoning is very harmful to various organs viz. heart, intestine and kidneys. Besides, it affects bones, tissues, nervous and reproductive systems. Hence, it is important to remove Pb (II) from aquatic environment. Polypropylene (PP) and polypropylene grafted-maleic-anhydride (PP-g-MA) based nanocomposites reinforced with Chitosan (CS) and modified montmorillonite clay nanofiller (CL120DT) were successfully fabricated using twin screw melt extrusion for adsorption of Pb (II). The resulting nanocomposites were characterized by XRD to analyze the dispersion properties of the material, TEM and SEM for surface morphology, FTIR analysis for the functional groups and TGA for thermal stability. Pure PP showed two sharp peaks, but there was decreased in the intensity upon adding of CS and CL120DT. Among series of nanocomposites 2.0 phr and 4.0 phr loaded samples shows better storage module than that of pure PP. The uptake of Pb (II) from lead nitrate aqueous solution by PP + PP-g-MA/CL120DT-CS 2.0 phr nanocomposites followed the Langmuir isotherm model, with a remediation of 90.9% at pH 8 and was verified by pseudo-second order kinetic model. These results indicate that PP + PP-g-MA//CL120DT-CS 2.0 phr nanocomposites performed as a superabsorbent for the Pb (II) ion removal from aqueous solution.

## Introduction

Heavy metal contaminants released into the environment from many industries, such as electroplating and metal plating facilities, metallurgical operations, welding, alloy manufacturing, fertilizer industry, agricultural activities and tanneries. The heavy metals found in traces levels in the aqueous stream pose a significant toxicity to the health and environment. Heavy metals tend to accumulate in natural biological systems and being non-biodegradable, these cause serious health disorders^1^. Thus, to remove hazardous heavy metals from water bodies and industrial wastewater has become a challenging task for researchers across the globe^2^. There are various techniques available for the removal of heavy metals form water environment; these include, adsorption, ion-exchange, chemical precipitation, membrane filtration, electrochemical technology and reverse osmosis^3–10^. Nevertheless, most of the treatment technologies are cost effective. However, among the above stated techniques, adsorption is one of the simple, easy to handle and low cost technique for removal of heavy metals.

Numerous adsorbents were studied for the removal of toxic inorganic elements from industrial waste water discharge such as activated carbon, fly ash, sawdust, crab shell, coconut shell^11–13^, silicates, natural zeolite, peat, chitosan polymer and biomass^2–14^. However, the adsorption capacity remains a major issue for currently existing adsorbents due to their low adsorption efficiencies for lower concentrations of heavy metal ions^13^ The need to develop novel nanomaterials with improved affinity is desired. Fortunately, polypropylene (PP)/modified clay-chitosan nanocomposites are providing an opportunity to solve the above mentioned problem. PP is the most important semi crystalline polymer materials with superior material properties such as high mechanical strength, porosity, thermal stability and melting temperature and therefore is most desired matrix among rest of the polyolefins to fabricate polymer nanocomposites^15,16^ hence it’s a good material for remediation if metal ions. Modified clays such as montmorillonite (MMt) is regarded as one the best inorganic adsorbent due to its, abundant availability, environmental friendly and a high negative^17,18^ charge which can adsorb positively charged metal ions. By incorporating a biopolymer such as chitosan into the composite, the surface area, would increase and therefore yield in the efficiency of the adsoprtion capacity. These nanocomposite show better material properties that otherwise cannot be obtained by conventional composites or pure polymers. Among hybrid materials, the mixtures of synthetic and natural polymers represent a simple way to combine their best properties, obtaining materials with acceptable morphological, thermal, and mechanical properties, compared to pure polymers^19^. However, to date, no studies have investigated the possibility of PP/modified clay-chitosan as a polymer clay nanocomposite for remediation of heavy metals in wastewater, surface water or even aqueous solutions.

Generally, three techniques are employed for polymer nanocomposites such as *in-situ* polymerization, solution blending, and melt mixing. Among them melt mixing is used to prepare polymer nanocomposites, which has environmental and economically advantages, due to the absence of solvents and monomers. In addition, melt extrusion is quick and easy to use. These are the main motivation for preparation of PP/modified clay-chitosan nanocomposites through melt extrusion. For this purpose, a good state of modified clay and chitosan dispersion within PP matrix is essential. We proposed an alternative approach for PP/modified clay-chitosan nanocomposite preparation. The effectiveness of this approach is examined here.

In this study, we report for the first time the synthesis of PP/modified clay-chitosan nanocomposite, prepared using micro-injection molding and melt-mix extruder techniques on the remediation of the Pb (II) heavy metal from aqueous solutions. The adsorption efficiency of the PP nanocomposite adsorbent was studies at different modified clay; modified clay-chitosan content, pH of aqueous solution, and contact time between Pb (II) and the nanocomposite. The analyzed adsorption data showed that the adsorption process of Pb (II) indicated pseudo-second order kinetic model. Furthermore, Characterization of prepared polymer composites is performed using FTIR, XRD, TEM, SEM, TGA and DMA techniques.

## Experimental

### Materials and methods

MMT types of clay were procured. For the study CL120 (China Glaze Co., NTC-34, and CEC: 168 meq/100 g) was used. (Dimethyloctadecyl [3-(trimethoxysilyl) propyl] ammonium chloride, DTSACl) [(CH_3_O_3_Si (CH_2_)_3_N (CH_3_)_2_(CH_2_)17CH_3_] Cl and Tetraethyl Orthosilicate (TEOS) were purchased from Sigma Aldrich. Polypropylene (PP) and Polypropylene grafted Maleated Anhydride (PPgMA) Maleic anhydride with the graft ratio 1 wt% were procured from LCY Chemical Corporation. Nitric acid (HNO_3_) schralan, Chitosan (CS), Hydrochloric acid (HCl) and potassium hydroxide (KOH) were purchased from Sigma, Ammonium hydroxide (NH_4_OH) pharmaco, Anhydrous Na_2_SO_4_ and Pb(NO_3_)_2_ were supplied by Merck Chemicals, South Africa. No further purification was done for all of these materials and were used as received.

### Synthesis and Modification of MMt clay (CL120)

The CL120 was dispersed in 60 mL of double distilled water using magnetic stirrer for time period of 24 h at room temperature to obtain swelled clay with the pH adjusted to 2~3 followed by the addition of DTSACl (0.834 g) solutions separately. Afterwards, and the mixture was allow to stirred for 1 h at 60 °C. After 1 h in each reaction mixture, the pH was adjusted to 5 and to this mixture TEOS (6.9 g) and ethanol (36 mL) solution was separately added and the combined mixture was heated at 70–80 °C for 24 h. This was later centrifuged and dispersed in deionized water, and the process repeated for 4–5 times. The modified clay was finally freeze-dried for 24 h. The obtained powders are represented by CL120-DT (modified MMT clay) respectively^20^.

### Preparation of PP-*g*-Ma/CS and PP-*g*-Ma/CS/CL120-DT nanocomposite

All modified clays, chitosan, PP and compatibilizer PPgMA used in our experiments were be dried at 100 °C in an air-circulating oven before compounding processes. This was done to prevent hydrolytic degradation. The CL120-DT-CS was blended with the PP, PPgMA, and nanofiller content were 2 and 4 phr with respect to PP and the compatibilizer PPgMA. The nanocomposites were prepared using melt-extruded in a laboratory scale (DSM twin screw micro compounder) at 180 °C (rotation speed 80 rpm, residence time 10 min). The nanocomposites prepared using above master batch approach are represented as PP + PPgMA/CL120DT-CS 2.0 phr, and PP + PPgMA/CL120DT-CS 4.0 phr.

### Adsorption studies

#### Swelling studies

The swelling properties of the nanocomposites were determined by immersing 0.05 g of nanocomposite sample dispersed in 15 ml distilled water over a period of time using variable pH values. The swelling [S%] of the nanocomposite was calculated using the following equation:

1 $$S \% =\frac{Mt-Mo}{Mo}100$$

where M_t_ is the mass of the swollen gel at time t and M_o_ is the dry gel at time 0.

#### Batch equilibrium studies

Stock solution of Pb(II) was prepared by dissolving lead nitrate salt using de-ionized water for a concentration of 100ppm. The adsorption study was carried out via batch adsorption experiments to evaluate the effect of pH, contact time and initial concentration and dose on Pb(II) adsorption. The weight of the adsorbent was kept constant at 0.050 g for all the experiments except for the studies on the effect of pH, and dosage where the weight was 0.150 g and the volume of Pb(II) solutions were fixed at 15 ml. 0.125 M NaOH or HCl was used to adjust the initial pH where as the initial concentration range of 10 ppm to 50 mg/l was used for the experiments. The solution was shaken thoroughly using a mechanical shaker with a rotating speed of 250 rpm. This was later filtered using a Whatmann filter paper at different time interval. The residual metal pollutant was determent by ICP-OES (Aligent 720 series)

The amount of metal adsorbed (qe) and percent of removal (%R) can be calculated using the following equations:

2 $${\rm{qe}}=\frac{({\rm{Co}}-{\rm{Ce}})}{{\rm{Vm}}}$$

3 $$ \% {\rm{R}}=\frac{({\rm{Co}}-{\rm{Ce}})}{{\rm{Co}}}100$$

where qe corresponds to the amount of metal pollutant adsorbed (mg/g), Co and Ce refer to the initial and equilibrium concentrations of the metal pollutant, respectively. V indicate the volume of the solution in liters, and m is assigned to the weight in grams (g) of the polymer nanocomposite.

#### Batch kinetic studies

Kinetic experiments were identical to equilibrium experiments except for the variation of time. Further, the remaining concentration after adsorption was converted to adsorption capacity by:

4 $${\rm{qt}}=\frac{({\rm{Co}}-{\rm{Ct}})}{{\rm{Ws}}}$$

where Ct refer to the residual concentration (mg/l) at time (t). The designation of the other variables have the been defined as in Eq. (3). The percent Pb(II) removed indicate as (R (%)) was calculated using Eq. (5).

5 $$ \% {\rm{R}}=\frac{({\rm{Co}}-{\rm{Ct}})}{{\rm{Co}}}100$$

### Characterization

X-ray diffraction was done using Bruker D8 for the advance detection of layered material with the scan angle covered at 2° < 2θ < 70° and the step size of 0.04°, 1 sec per step. The morphology and the internal structure of PP nanocomposites was determined using Transmission electron microscopy (TEM) on a Model JEOL JEM2010, 200 kV. A sample with a thickness of 80 nm was prepared using a Leica Ultracut-UCT in order to study the physicochemical properties of nanocomposites. Thermal stability of the nanocomposites were evaluated using SII TG/DTA6200, using 10 mg sample of PP nanocomposites under air gas flow in the temperature range of 40 °C to 900 °C at a scanning rate of 10 °C min^−1^. TA-Q800 instrument was used to analyse the dynamic mechanical analysis (DMA) measurement that was carried out for the sample size: 35 mm * 12 mm * 3 mm.in the air at a scanning temperature range of 30–210 °C at a heating rate of 5 °C min^−1^. Tensile strength was evaluated with Tensile Testing Machine (Come tech D638 instrument). Analysis of metal trace was achieved by Inductive coupled plasma (ICP-OES) model 720 series.

## Results and Discussion

### Characterization of clay and modified clay

**Figure 1:**
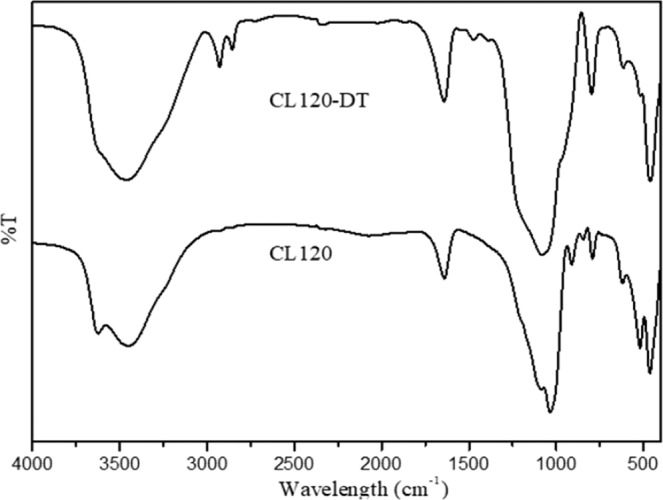
FTIR spectra of pure and modified clay.

**Figure 2:**
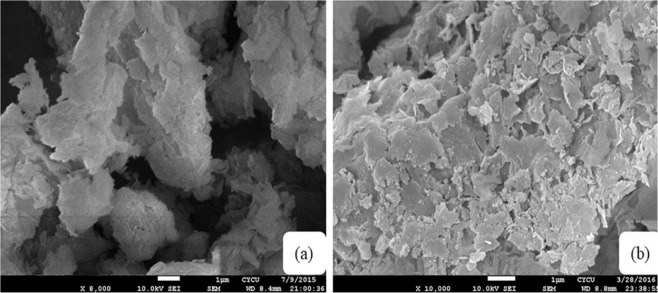
SEM images of (**a**) CL120, (**b**) CL120-DT.

Fourier transform infrared (FTIR) spectra for the identification of functional groups of the modified clays are shown in (Fig. 1). The spectra show a broad between 3100 cm^−1^ and 3600 cm^−1^ that can be assigned is to an O–H stretching vibration of the hydroxyl group and interlayer water. The bands at 2930 cm^−1^, 2867 cm^−1^ and 1470 cm^−1^ were observed that may be attributed to asymmetric and symmetric aliphatic alkyl chain stretching,. The peaks at 460 cm^−1^ and 520 cm^−1^ can be designated to the vibration bands of Mg-O and Al-O, respectively. The viberational bands of Si-O-Si in the brucite like metal hydroxide layer were observed at 991 cm^−1^,1025 cm^−1^, 1045 cm^−1^, 1084 cm^−1^ and 1108 cm^−1^. All modified clays showed vibration bands characteristic of modifier functional groups determined the electrostatic interaction with anionic clay layers. In addition, the surface morphology of the modified clays were studied using SEM analyses. As shown in (Fig. 2), CL120, and CL120-DT have an aggregated plate like morphology. However, the modification of the clay showed that the surface had an irregular petal shape along with and non-laminar stacking (Fig. 2)^16^.

### Morphology of PP nanocomposites

**Figure 3:**
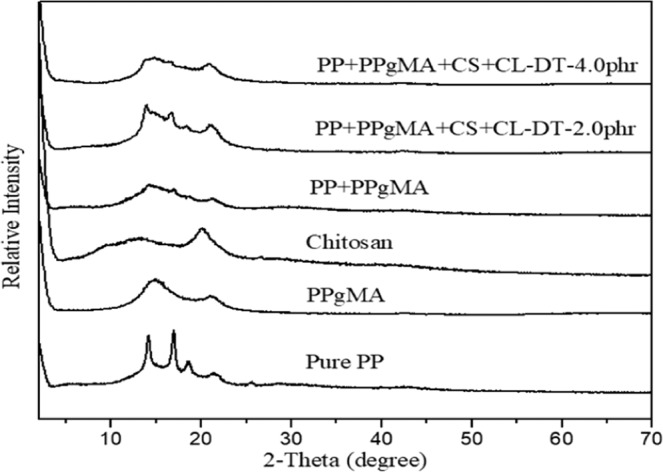
XRD pattern of PP and PP nanocomposites.

**Figure 4:**
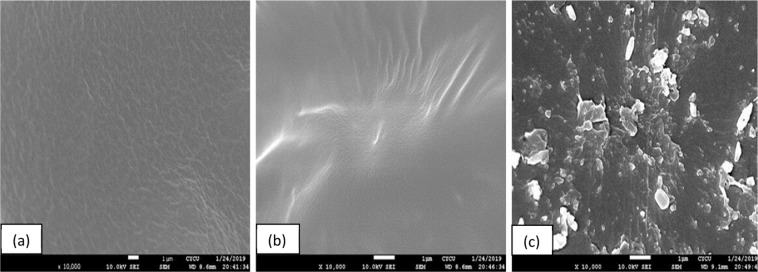
SEM images of (**a**) PP (**b**) PP + PPgMA and (**c**) PP + PPgMA + CS-CL-120dt 2phr nanocomposites.

The XRD patterns of pure PP, and PP nanocomposites containing 2.0 phr, and 4.0 phr CL120DT-CS are displayed in (Fig. 3). Pure PP showed two sharp peaks, PP nanocomposites intensity peaks of PP decreased by addition of chitosan and clay corresponding to a more disordered structure due to a decrease in the degree of coherent layer stacking. Also, characteristic CL120DT peaks were not found after the formation of PP nanocomposites by melt extrusion, it is due to well stacked clay layers exfoliated or intercalated in the PP matrix. The dispersion morphology of modified clay and chitosan in the PP matrix was further verified by scanning electron micrographs micrographs of PP and PP nanocomposites shown in (Fig. 4) where the surface shows a fairly homogenous polymer. The images show the effects of PPgMA and Chitosan content on PP composites. (Fig. 4(c)) shown smaller particles on surface those are indicate the Chitosan particles. It is evident that PP is a non-polar polymer and has a poor bonding ability with the filler. Thus, PPgMA help and to act as the bridge between PP and filler after addition of Chitosan the surface roughness increased as shown (Fig. 4c). When the chitosan content increased further effect the thermo-mechanical and absorption properties.

**Figure 5:**
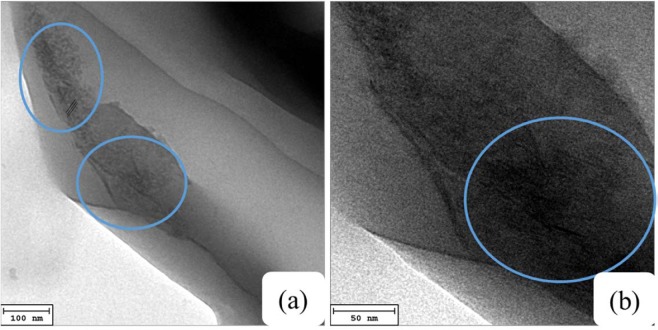
TEM images of PP + PPgMA/CL120DT-CS 2.0 phr nanocomposites with different magnification.

**Figure 6:**
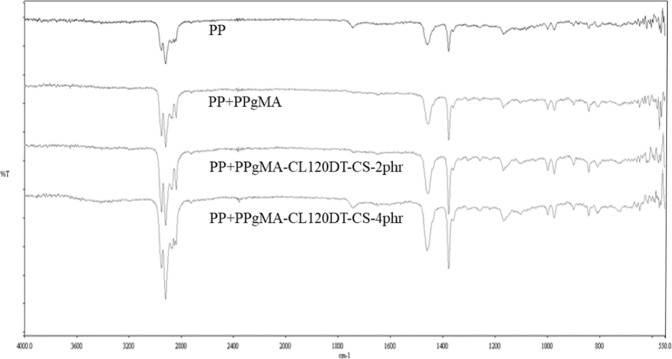
FTIR spectra of polymer nanocomposites.

The transmission electron micrographs of PP nanocomposites are presented in (Fig. 5) showing the cross-sectional morphologies indicating exfoliated modified clay layers that were well dispersed in the PP matrix. Horizontal lines in the TEM images indicate the clay nanosheets whereas the dark line indicate the cross-section of nanolayer. It was observed that the degree of delaminated platelets along with the dispersion of nanofiller particles are comparatively low and some clay stacked layers still exist in the polymer matrix as shown in Fig. 4(b).

(Figure 6) presents the spectra of the PP in comparison with the samples nanofilled with 2 and 4 phr of mass fractions of CL120DT-CS. All the spectra present the characteristic peaks of PP: –CH2 and –CH_3_ stretching vibrations (2800–2950 cm^−1^), –CH_3_ and –CH_2_ bending (1376, 1456 cm^−1^) and C–CH_3_ stretching (841 cm^−1^).

### Thermo-Mechanical Properties of PP Nanocomposites

**Figure 7:**
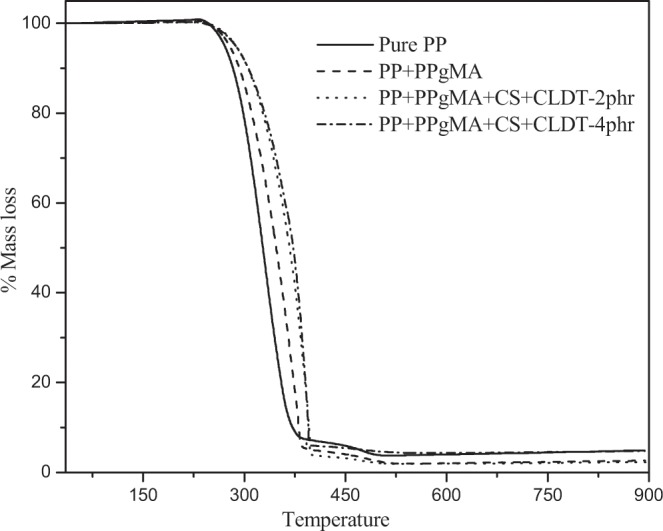
TGA curves of PP nanocomposites.

TGA thermograms were based on weight loss temperature (T_5d_) (5%) of PP nanocomposites, thermal stability is decreased as compare to pure polymer results summarized in Table 1 and (Fig. 7). There is a decrease in the thermal stability that can be that is probably due to the homogeneous dispersion of the chitosan in the PP matrix and chitosan particles is expected to settled in-between the polypropylene polymer chains with a high interfacial contact area. T_5d_ of pure PP is 280 °C and chitosan decomposition starts from 200 °C, this might be the reason to decrease the T_5d_ with the chitosan content.

**Table 1:** Thermo-mechanical properties of PP nanocomposites.

Sample	T_5d_ (°C)	Storage module (Mpa)	Tcc (°C)	Tm (°C)
PP	280 ± 2	1752	118.49	167.22
PP + PPgMA	278 ± 4	1700	118.89	167.50
PP + PPgMA CS + CL120DT-2phr	273 ± 3	1767	118.49	167.72
PP + PPgMA CS + CL120DT-4phr	277 ± 5	1833	118.49	167.72

The storage modules of the pure PP and PP nanocomposites was evaluated by DMA analysis, the selected sample results summarized in Table 1. It is observed that the PP/CL120-DT-CS nanocomposites storage modulus increased when compared to that of the pure PP. Increasing the content of the CL120-DT-CS leads to an increase in the thermal stability that occur due to the exfoliation of clay platelets bye the polymer chains. The structure of the composites as determined from XRD indicated intercalation and delamination of clay platelets that had an impact on overall thermal stability.

**Figure 8:**
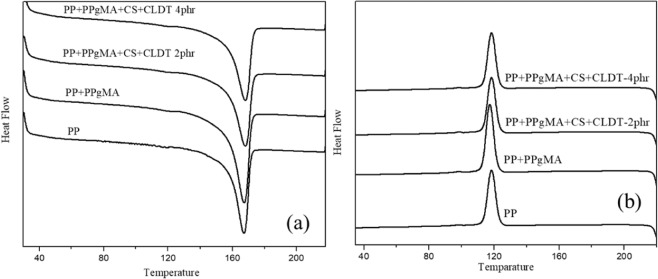
DSC curves of Heating and cooling polymer nanocomposites.

The DSCs (Fig. 8) of the pure PP and PP nanocomposites results are summarized in Table 1. The melting peaks (Tm) as well as the cooling crystal temperature (T_CC_) of all the nanocomposites were nearly similar regardless of the amount of CL120DT-CS, referring to the fact that the addition of clay does not alter the crystal structure of the PP matrix after the formation of nanocomposites.

### Heavy metal uptake

**Figure 9:**
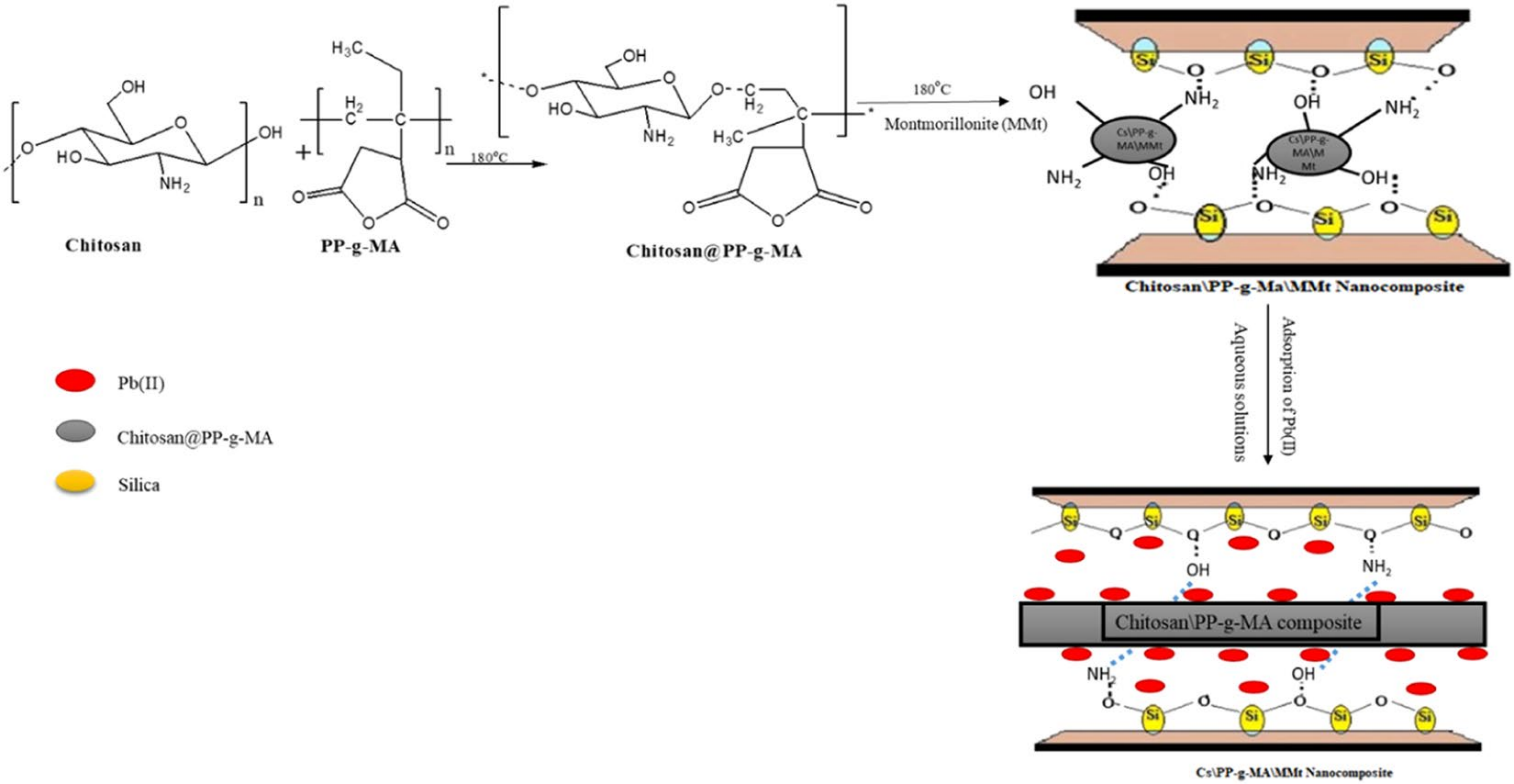
Mechanism of PP-g-MA\CS\Cl (MMt) illustrating the possible bonding and the uptake of lead ion from aqueous solution.

(Figure 9) below shows the possible bonding mechanism between the PP-g-MA\Cs\Cl (MMt) nanocomposite and Pb (II). As illustrated below, chitosan is modified with PP-g-MA, through covalent bonding. As a result a composite is formed with improved characteristics for adsorption of Pb (II) ions. Chitosan has N-H, and excess OH^−^ functional groups and PP-g-MA is highly porous, hence a good combination for adsorption processes. Due to the high negatively charged surface of clay materials, MMt clay nanoparticles are incorporated into the composite to further enhance the composite properties. Therefore Cs acts as a bridging component between the MMt clay and PP-g-MA, as shown in Fig. 8. At pH 8 the adsorption of Pb (II) reaches optimal, this may be due to the increase of OH^-^ (hydroxyl ions) but slightly decreases till pH 10 as shown in Fig. 8. Then a major drop is observed at pH > 10. This might be attributed to the precipitation of Pb (II) at pH ∼ 8.4. Hence, the sudden uptake of Pb (II) by PP-g-MA\CS\Cl at pH < 7 is not attributed to the formation of Pb (OH)_2_. In conclusion, the results in Fig. 8, indicate that the optimum pH values of the binary system of the polymer blends to remove Pb (II) from solution by using PP-g-MA\CS\Cl are 8–10.

#### Effect of Swelling

**Figure 10:**
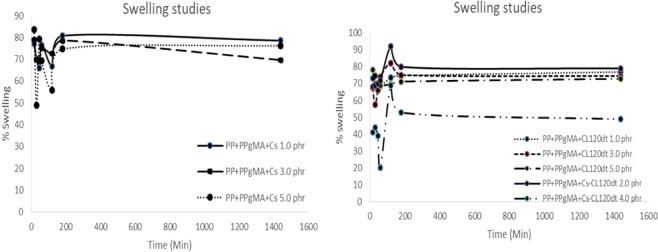
Swelling capacity of PP + PP-*g*-MA based nanocomposites in deionized water.

Swelling behavior of the composite hydrogels with polymer/clay structure were investigated for PP, PPgMA, CL and CS nanocomposite with different %Wt. illustrated in (Fig. 10). According to Ren *et al*. 2011, the swelling conditions of nanocomposites gels exhibits characteristics of swelling and deswelling behavior such as initial large swelling, maximum swelling and followed by the subsequent deswelling towards an equilibrium state. In (Fig. 10(a)), PP + PPgMA\CS with different %Wt ratio (1.0, 3.0, 5.0 phr) respectively was introduced to distilled water for analyzing the swelling capacity of the polymer composite. The sample was measured for 15 to 1440 min. The analysis indicated a gradual increase in the swelling studies (initial large swelling) and reaches maximum equilibrium at 120 minutes, thereafter a gradual decrease is observed, this may be due to the deswelling of the nanocomposite. At 15 min the swelling percentage of PP + PPgMA/CS 1.0phr reaches optimum at 85%. Therefore, upon incorporation of CL120DT nanoparticle to PP + PPgMA/CS, illustrated in (Fig. 10(b)), the nanocomposite (PP + PPgMA/CL120DT-CS 2.0 phr) shows gradual increase and reaches max at 92% of swelling capacity at 120 min. These may be due to the increase in the surface area of CL120DT and the ability of the nanoparticle to swell. Thereafter, from 120 to 180 min the nanocomposite is observed to decreases drastically in swellability by almost 10%. An improvement in water solubility might be due to the solubility of the complex that occur during reinforcement of the polymer blend (PP + PPgMA/CS) by the clay (CL120DT). Therefore, due to the swellability of PP + PPgMA/CL120DT-CS 2.0 phr nanocomposite reaching maximum amount. The kinetics models and adsorption studies will be based on the PP + PPgMA/CL120DT-CS 2.0 phr nanocomposites

#### Effect of pH and concentration

**Figure 11:**
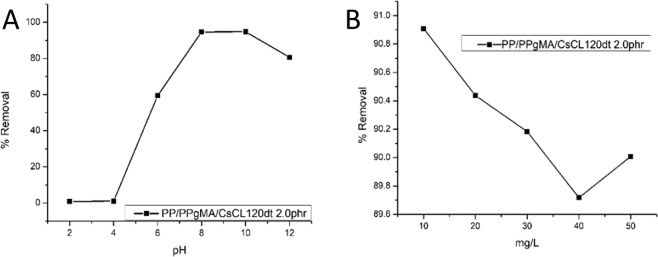
(**A**) Effect of pH on the adsorption of Pb (II) by PP + PPgMA + CS-CL120DT 2.0 phr nanocomposite. (**B**) Concentration dependent performance characteristics PP + PPgMA + CS-CL120DT 2.0 phr nanocomposite, on Pb (II) adsorption.

pH of the solution affects the overall surface charge of the adsorbent (PP + PPgMA/CL120DT-CS 2.0 phr) and the charge on the adsorbate Pb (II). The results of pH variation showed that maximum metal removal was found at pH 8 with an accumulation off 90.9% as shown in (Fig. 11A) and was previously^21^. From the pH range of 2.0–4.0, the *q*_e_ shows no changes but there is a drastic change from pH 4.0–10.0, this is due to pH_PZNC_ value of the montmorillonite sample at high pH. The study indicated that the overall negative charge density of the adsorbent increased with the pH, which triggers he initial sharp increase of the *q*_e_ and after pH 4.0 it approaches adsorption equilibrium. The mechanism of pH dependence of Pb(II) ions uptake can be explained by the nature of composite surface – metal binding sites based on the study of Pb (II) adsorption by mixed fly ash^22^, pointed out that heavy metal ion adsorption could be divided into stages, precipitation or hydrolyses^8^. The ability of PP + PPgMA/CL120DT-CS 2.0 phr to adsorb Pb(II) is attributed to the presence of ions, as OH^−^, and NH^2+^ groups and the highly negative surface of CL120DT, which could attract positively charged (M^+^)^23^. Figure 8a indicate adsorption or superficial deposition of Pb (II) onto the adsorbent^21^. To study the effect of initial concentration by PP + PPgMA/CL120DT-CS 2.0 phr composites, five different initial concentrations of Pb (II) were chosen viz. 10, 20, 30, 40 and 50 mg/l with a constant dose of 0.05 g of adsorbent nanocomposite and the results are shown in (Fig. 11B). The ability of PP + PPgMA/CL120DT-CS 2.0 phr to adsorb Pb (II) is attributed to the presence of active sites such as OH^−^, and NH_2_^+^ that enhances the swelling capacity. The PP + PPgMA/CL120DT-CS 2.0 phr polymer composite was able to uptake 90.9% of the Pb (II) at a 10 mg/l and a constant dose of 0.05 g^24^. However, there was a no significant change in adsorption of Pb (II) in all concentrations.

#### Effect of dose and time

**Figure 12:**
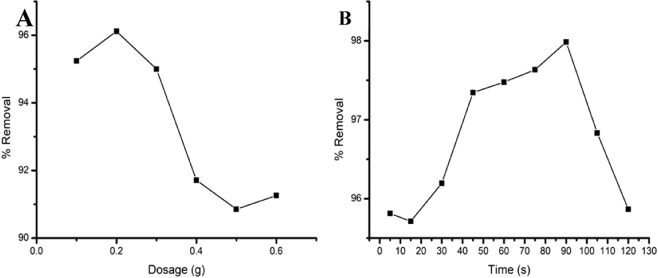
(**A)** Effect of adsorbent dosage on Pb(II) ion by PP + PPgMA + CS-CL120dt 2.0 phr nanocomposite. (**B)** Time dependent performance characteristics PP + PPgMA + CS-CL120dt 2.0 phr nanocomposite, on Pb (II) adsorption.

The adsorbent dosage is an important parameter for adsorption and kinetics efficiency during treatment at a wastewater treatment. (Fig. 12A) illustrate removal efficiency over a variation of dose (0.1 to 0.6 g). The figure clearly shows that the adsorption efficiency of Pb(II) raised from 92 to 94% in the range of dosage from 0.1 g to 0.2 g at 30 min that would be due to the larger surface area providing more active sites for adsorption. However, from 0.2 g onwards there is a minor drop of the % removal, the sudden drop at 0.2 g which can be attributed to a cluster of competing ions available in the nanocomposite, the drop of efficiency can amount to about 4.0%. Hence the optimal removal efficiency was achieved at 0.2 mass with 94% efficiency. (Fig. 12B) below, shows adsorption kinetics for the removal of Pb (II) over a period of time range (5, 15, 30, 45, 60, 75, 105, 120, min) using 0.2 g of PP-g-MA/Cs/CL 120dt 2.0phr nanocomposites pH 8 at a concentration of 10 mg/l initial concentration. From Fig. 11B it was analyzed that maximum removal of Pb (II) had 98% at 90 min contact time as reported earlier^25^.

#### Effect of time on adsorption capacity

**Figure 13:**
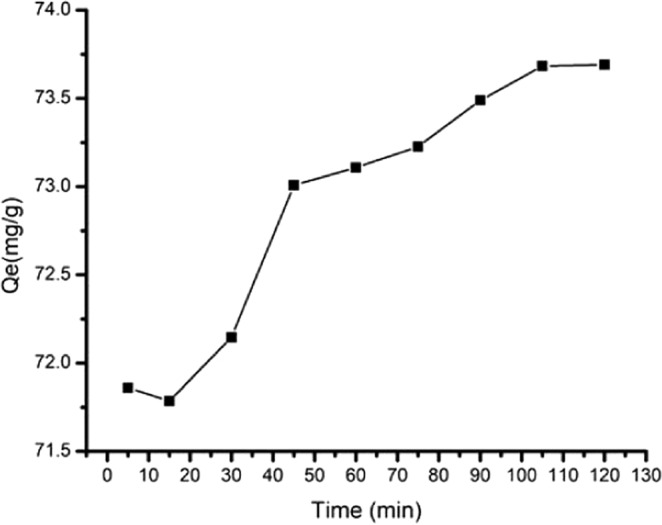
Effect of time on adsorption capacity of Pb(II) ion by PP + PPgMA + CS-CL120dt 2.0 phr nanocomposite.

The adsorption kinetics was carried out with variation in adsorption times on the PP + PPgMA + CS-CL120dt 2.0 phr nanocomposite to remove Pb (II) are shown in Fig. 12. With the increase in the time, the Pb (II) adsorption capacity of the PP + PPgMA + CS-CL120dt 2.0 phr nanocomposite initially decreased but suddenly increased rapidly to reach equilibrium. The maximum capacity adsorbed was at rate of 73.8 mg/g illustrated in Table 2. Pb (II) ions were adsorbed by the presence of ions, as OH^−^, and NH_2_^+^ groups and the highly negative surface of CL120dt, which could attract positively charged (M^+^). Therefore, (Fig. 13) indicated adsorption or surficial deposition of lead ions diffusing into the microporous adsorbent forming a complex within the active sites of the adsorbent.

**Table 2:** Comparison studies of different adsorbents for adsorption for Pb (II) and other metal ions.

Adsorbent	Metal ion	Adsorption capacity (mg/g)	Isotherm	References
Poly-analine grafted chitosan	Pb (II)	16 mg/g	Langmuir	^24^
PP-PP-g-MA/Cs/CL120dt	Pb (II)	73.8 mg/g	Langmuir	Present work
MMMT@Zn-BDC	Pb (II)	724.64 mg/g	Langmuir and Freundlich	^28^
CTS-MMT) composite beads	Ag(I)	43.48 mg/g	Langmuir	^29^
MMT-MSA	Pb(II)	74.7 mg/g	Langmuir	^30^
Dextrin/MMT	Pb(II)	284.593 mg/g	Langmuir	^31–33^

### Adsorption isotherms

#### Langmuir Isotherm

Two isotherm models viz Langmuir, and Freundlich were evaluated to determine the distribution of Pb(II) ions between the adsorbate and the adsorbent phase when equilibrium was obtained. The equation for Langmuir isotherm is given by Eq. (6) below

6 $$\frac{1}{{\rm{qe}}}=\frac{1}{{\rm{QK}}}+\frac{1}{{\rm{KQCe}}}$$

where, Ce is the equilibrium concentration of the adsorbate (lead solution) (mg/L), qe the amount of adsorbate adsorbed per unit mass of adsorbate (mg/g), and Qo and b are the Langmuir constants related to the monolayer adsorption capacity. Langmuir constants ‘b’ and ‘Qo’ were calculated from this isotherm and their values are given in Table 2.

**Figure 14:**
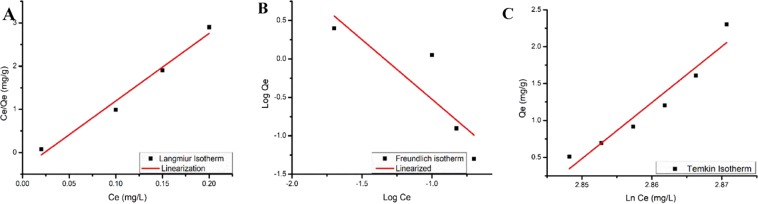
(**A**) Linearized fits for Langmuir isotherm 10 mg/l of Pb (II), 0.2 g of by PP + PPgMA/CL120DT-CS 2.0 phr composite, (**B**) Linearized fits for Freundlich isotherm 10 mg/l of Pb (II), 0.15 g of PP + PPgMA/CL120DT-CS 2.0 phr composite, (**C**) Linearized fits for Temkins isotherm 10 mg/l of Pb (II), 0.2 g of PP + PPgMA/CL120DT-CS 2.0 phr composite.

In this study, a plot of Ce/qe versus Ce produced a straight line with a correlation coefficient (R^2^ = 0.99), confirming that the lead ion adsorption onto PP + PPgMA/CL120DT-CS 2.0 phr polymer composite follows Langmuir isotherm as given in (Fig. 14A)^26^. The separation factor RL defined by Eq. 7, was found to be 0.998.

7 $${\rm{RL}}=\frac{1}{1+{\rm{K}}Co}$$

where Co is the optimum initial concentration (mg/L) of metal ions and K is related to the energy of adsorption (L/mg)

#### Freundlich Isotherm

The Freundlich isotherm model is analyzed from the Eq. (8) below:

8 $$qe=KfC{e}^{1/n}$$

Logarithmically Eq. (8) above can be further modified to Eq. (9):

9 $$\log \,qe={\rm{logK}}f+\frac{1}{n}\,\log \,Ce$$

In the above equation, *Kf* and *n* are designated as the Freundlich constants. A linear curve was obtained from the plot of log *Ce* vs. log *qe* providing an intercept value of *Kf* indicating the adsorption capacity of the adsorbent and the slope of *n* showing the adsorption intensity^27^. Freundlich isotherm model is commonly used indicate if the adsorption is dependent or independent of pressure whereas Langmuir isotherm indicate on the monolayer adsorption capacity. In this study, the constants K_f_ and n were derived from the intercept and slope respectively from the plot of log Ce against log qe.

The Freundlich constants K_f_ and n were found to be 0.046 mg/g was evaluated from the (Fig. 14B). The isotherm data does not fit the Freundlich model well (R^2^ = 0.7554) and the non-linear plot indicates that the adsorption of Pb (II) ions onto polymer composites does not follow the Freundlich isotherm. Also, the slope 1/n indicates the effect of concentration on the adsorption capacity and various values from all sorption measurements are illustrated in Table 3 below.

**Table 3:** Langmuir model, Freundlich, Temkins model results of PP nanocomposites.

Sample	Langmuir model		Freundlich model					Temkin model			
	Q_o_	K	R_L_	R^2^	K_f_	N	R^2^	A_t_	Bt	b	R^2^
Pb (II)	42	0.64	0.61	0.99	4.71	0.65	0.7554	1.031	75	32	0.9327

#### Temkin isotherm

In (Fig. 14C) Temkin Isotherm is shown. The Temkin isotherm is used to evaluate the heterogeneous surface energy systems indicating non-uniform distribution of sorption heat and take into the account of adsorbent–adsorbate interactions. Tempkin model defines a linear reduction of heat of adsorption ΔH (as a function of T in kelvin) of all particles in the layer that refers to a uniform distribution of binding energies (up to some maximum binding energy). This was carried out by plotting the quantity adsorbed qe against ln Ce and the constants were evaluated from the slope and intercept. The following equation below represents the Tempkin model; where AT is Temkin isotherm equilibrium binding constant measured in L/g, Bt are Temkin isotherm constant, R is universal gas constant (8.314 J/mol/K), T is Temperature at 298 K and lastly B is Constant related to heat of sorption(J/mol)

10 $$qe=\frac{{\rm{RT}}}{{\rm{B}}}{\rm{lnK}}tCo$$

The linearized Eq. (10) above can be transformed to Eq. 11 below

11 $$qe=\frac{{\rm{RT}}}{{\rm{B}}}{\rm{lnK}}t+\frac{{\rm{RT}}}{{\rm{B}}}\,\mathrm{ln}\,Ce$$

From the plot in (Fig. 14C), Temkins calculations are estimated; where: A_T_ = 1.031 L/g, B_T_ = 75.991J/mol refers to the heat of sorption indicating a physio sorption and the value of **R**^**2**^ was **0.9327**.

### Kinetic model

#### Pseudo 1^st^ order

Pseudo-first-order and pseudo-second-order kinetic models were employed to understand the mass transfer and rate of reactions for the adsorption of Pb (II) metal ion onto the strip (PP + PPgMA/CL120DT-CS 2.0 phr polymer composites).

**Figure 15:**
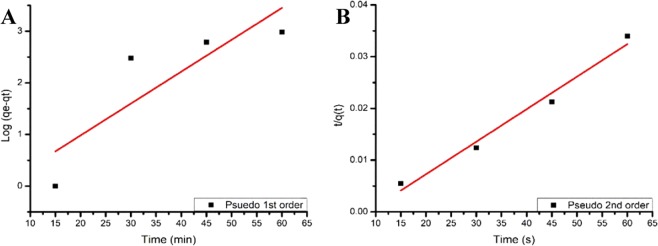
(**A**) The pseudo-first-order kinetics for the adsorption of Pb^2+^ onto PP + PPgMA/CL120DT-CS 2.0 phr polymer composite. (**B)** The pseudo-second-order kinetics for the adsorption of Pb (II) onto PP + PPgMA/CL120DT-CS 2.0 phr polymer composite.

The linear form of Lagergren *et al*., equation as illustrated in Eq. (12) below was used to evaluate the pseudo-first-order

12 $$\log (qe-qt)={\rm{logqe}}\frac{{\rm{Kad}}}{2.303}{\rm{t}}$$

where qe and qt refer to the amounts of Pb(II) (mg/l) adsorbed on PP + PPgMA/CL120DT-CS 2.0 phr polymer composite at equilibrium time and time t (h) and K_ad_ referring to the pseudo-first-order constant (min^−1^). The rate constant, K_ad_ and correlation coefficients for different concentrations of the Pb(II) ions were calculated from the linear plots of log (qe-qt) versus t. A best fit line was plotted and the very poor correlation coefficients (R^2^) were obtained as shown in (Fig. 15A) indicated that the sorption of Pb(II) ions onto PP + PPgMA/CL120DT-CS 2.0 phr polymer composite could not satisfy pseudo-first order kinetics.

#### Pseudo 2^nd^ order

The pseudo-second-order equation is expressed as:

13 $$\frac{t}{qt}=\frac{1}{K2q{e}^{2}}+\frac{t}{qe}$$

where h ¼ kqe^2^ (mg/g min) and k (g/mg min) is the pseudo second- order rate constant of adsorption. It is presumed that the plot of t/q versus t showing a linear relationship primarily. Figure 14B the plot for pseudo-second-order model. The linear fit (correlation coefficient, (R^2^ = 0.9806) show that the adsorption follows the pseudo-second-order model. The correlation coefficients for the second-order kinetic model were found to be greater than 0.9 showing the second-order nature of the adsorption process of Pb(II) ions onto PP + PPgMA/CL120DT-CS 2.0 phr polymer composite.

It is to be noted that the 2^nd^ order kinetics is only applicable if the plot of t/q(t) versus t shows a linear relationship. Figure 14B shows the plot for pseudo-2^nd^ order model. The linear fit with correlation coefficient, R^2^ = 0.9808 for Pb (II) indicating the pseudo-second-order model^27^. The correlation coefficients for the second-order kinetic model were found to be greater than 0.9 referring to the second-order nature of the adsorption process of Pb (II) on PP + PPgMA/CL120DT-CS 2.0 phr polymer composite. It was concluded that the Langmuir isotherm fitted the experimental data better when compared to the Freundlich and for Pb ions removal onto PP + PPgMA/CL120DT-CS 2.0 phr polymer composite depend on the value of the correlation coefficient R^2^.

## Desorption studies

**Figure 16:**
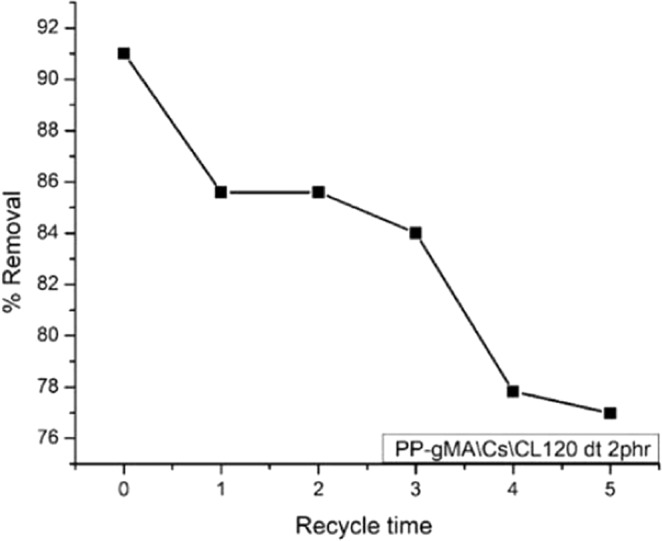
Effect of cycle time on Pb (II) desorption.

(Figure 16) shows the desorption studies of Pb (II) by PP-*g-*MA/Cs/CL120 dt 2phr in aqueous solutions. The purpose of desorption is to reuse and recycle the adsorbent for n number of cycles. Therefore, the repeated cycles of adsorption and desorption after regenerations of the adsorbent are significant indexes. The adsorption test were conducted at 25 °C, pH 8.3, initial Pb (II) concentration of 10 ppm, 0.5 g mass, 15 ml volume and 30 minutes contact time. The desorption experimentation were conducted at 25 C, pH 8.3, 1.0 NaNO_3_ desorption solutions, 10 ppm initial Pb(II) concentration, 30 min contact time. The desorption removal rate of Pb(II) concentration on PP-*g-*MA/Cs/CL120dt 2phr drastically decrease with increase of recycle time that lead to an early equilibrium resulting in increase in the residual Pb(II) ions concentration in solution with the increase in the number of the cycles. This was evident when the removal rate decreases from 82% to 77% as illustrated in the (Fig. 16) after three cycles below.

The gradual decrease performance might be due to the exchangeable ions available in CL120dt, and residual Pb(II) ions occupied by adsorption sites. The experimental results showed that PP-*g-*MA/Cs/CL120 dt 2phr maintained a stable performance on the desorption studies.

## Conclusion

**PP + PP-*g*-MA/CL120DT-CS** nanocomposite was successfully synthesized using melt mix extrusion. For molding, the samples were introduced to Mucell ® injection technique. The instrumental analysis of nanocomposite illustrates a decrease in the intensity after adding and **CL120 DT** onto **PP + PP-*g-*MA**, which was observed using **XRD**. The modified clay layers were well dispersed and exfoliated in the PP matrix as confirmed by **TEM**. Thermal stability of nanocomposites decreased as compare to pure **PP + PP-*g*-MA**. The decrease of thermal stability was assigned to the homogeneous dispersion of the **CS** with a high interfacial contact area between the **PP** chains. Confirmed by **TGA**. **PP + PP-*g*-MA/CL120DT-CS** nanocomposite was used for adsorption of **Pb (II)** metal ions from aqueous solutions. The factors such as pH and initial concertation affects governed the adsorption of **Pb (II)**. The nanocomposites were found to remove 90.9% of **Pb (II)** from the synthetic solutions. The removal of **Pb (II)** by **PP + PPgMA/CL120DT-CS 2.0 phr** polymer composite followed the Langmuir isotherm model that confirmed that **Pb (II)** ions was adsorbed on homogeneous sites. These homogenous active sites had uniform binding energies that allowed the formation of monolayer on the surface of the nanocomposite following pseudo-second order kinetics establishing an overall chemisorption of **Pb (II)** from aqueous solution.
